# Missing Components of Receptor Status Among Women With Invasive Breast Cancer

**DOI:** 10.1001/jamanetworkopen.2023.30791

**Published:** 2023-08-24

**Authors:** Julie A. Stephens, James L. Fisher, Robert Wesolowski, Electra D. Paskett

**Affiliations:** 1 Center for Biostatistics, Department of Biomedical Informatics, College of Medicine, The Ohio State University, Columbus; 2 Arthur G. James Cancer Hospital and Richard J. Solove Research Institute, Columbus, Ohio; 3 Division of Medical Oncology, College of Medicine, The Ohio State University, Columbus; 4 Comprehensive Cancer Center, The Ohio State University, Columbus; 5 Division of Cancer Prevention and Control, Department of Internal Medicine, College of Medicine, The Ohio State University, Columbus; 6 Division of Epidemiology, College of Public Health, The Ohio State University, Columbus

## Abstract

**Importance:**

There are well-known differences in patient outcomes and effective therapeutic options across subtypes of breast cancer (BC), defined by the status of estrogen receptor, progesterone receptor, and erb-B2 receptor tyrosine kinase 2 (*ERBB2 *[formerly *HER2*]) expression, making testing for these receptors part of the routine workup for all patients with a diagnosis of invasive BC. Despite its importance, this information is missing in some BC cases.

**Objective:**

To identify female patients with BC without record of testing for estrogen receptor, progesterone receptor, or *ERBB2* status, defined as missing components of receptor status (MCRS).

**Design, Setting, and Participants:**

This cross-sectional study used data from National Cancer Institute’s Surveillance, Epidemiology and End Results Program of 18 population-based registries from women with a diagnosis of invasive BC (excluding death certificate–only and autopsy cases) from January 2012 to December 2016. The final analyses were completed in February 2022.

**Main Outcome and Measure:**

The primary outcome was MCRS. Those with MCRS were summarized by age, race, stage at diagnosis, reporting source, primary payer, and geography. Multivariable logistic regression was used to estimate adjusted odds ratios (aORs) for MCRS.

**Results:**

Overall, 321 913 patients with invasive BC were included (1928 [1%] American Indian or Alaska Native, 28 173 [9%] Asian or Pacific Islander, 36 357 [11%] Black, and 252 447 [78%] White individuals); of these, 15 250 (4.7%) had MCRS. The multivariable model showed that the odds of MCRS were higher in women 80 years or older compared with those younger than 49 years (aOR, 1.75; 95% CI, 1.65-1.88), Black compared with White women (aOR, 1.09; 95% CI, 1.00-1.16), and those with distant stage or unknown/unstaged cancer at diagnosis compared with a local stage at diagnosis (aOR, 3.33; 95% CI, 3.17-3.50; and aOR, 19.39; 95% CI, 18.15-20.72; respectively). With hospital inpatient/outpatient or clinic as the reference group, cases reported by laboratory only, nursing/convalescent home/hospice, and a physician’s office were more likely to have MCRS (aOR, 1.42; 95% CI; 1.28-1.60; aOR, 9.37; 95% CI, 6.03-14.53; and aOR, 2.32; 95% CI, 2.06-2.62; respectively). Adjusted odds of MCRS were higher for the categories of insured/no specifics and insurance status unknown compared with those who were insured. The adjusted odds of MCRS were higher in rural compared with urban areas (aOR, 1.08; 95% CI, 1.03-1.15).

**Conclusions and Relevance:**

The results of this cross-sectional study of women with a diagnosis of invasive BC suggest that despite a standard of care recommended by all expert guidelines, there needs to be greater focus on hormone receptor and *ERBB2* testing in all women with invasive BC. The results of this study may help clinicians, public health practitioners, and policymakers target affected populations to minimize or eliminate this critical health disparity and help save more lives.

## Key Points

**Question:**

Do missing components of receptor status vary in the US by patient or disease characteristics?

**Findings:**

In this cross-sectional study of 321 913 patients with invasive breast cancer, the rate of missing components of receptor status was higher in older women, Black women, and women from rural areas, as well as those with unstaged cancers, cases reported from facilities other than a hospital, and unknown insurance status.

**Meaning:**

The results of this cross-sectional study suggest that there is a need to target some populations to ensure all individuals with invasive breast cancer are getting the testing needed to get the most accurate prognosis and treatment plan.

## Teaser

This cross-sectional study assesses the proportion of female patients with invasive breast cancer who have no record of testing for estrogen receptor, progesterone receptor, or *ERBB2* status.

## Introduction

An estimated 297 790 women will receive a diagnosis of breast cancer in the US in 2023, and nearly 43 170 women will die of the disease.^1^ Assessment of possible expression of estrogen receptor (ER), progesterone receptor (PR), and erb-B2 receptor tyrosine kinase 2 (*ERBB2 *[formerly *HER2*]) is standard in identifying the most suitable breast cancer treatment and determining prognosis. Breast cancer is grouped into 3 subtypes: hormone receptor positive and *ERBB2* negative (approximately 65%), *ERBB2* amplified (approximately 5%-20%), and triple negative (approximately 5%).^2^ The characterization of subtypes has changed how patients with breast cancer are treated.^3^ Because of this, testing for ER, PR, and *ERBB2* expression is the recommended standard of care for all of those with a diagnosis of invasive breast cancer.^4^

Reporting of ER and PR data to the National Cancer Institute’s Surveillance, Epidemiology and End Results (SEER) Program began in 1992. The proportion of cases with unknown ER and PR dropped considerably in 2003 when this information became required by Commission on Cancer–approved hospitals.^3^ Collection of *ERBB2* data started in 2010, with the proportion of unknown *ERBB2 *decreasing steadily until 2016 (Figure). From 2012 through 2016, the proportion of missing *ERBB2* was approximately 63% of higher than the proportion missing ER and PR (Figure). Missing ER, PR, and/or *ERBB2* status is associated with biases in estimating SEER incidence rates and survival probabilities for specific breast cancer subtypes. Howlader et al^3^ reported that *ERBB2* is not missing entirely at random varying by age, stage, race and ethnicity, county-level socioeconomic status, and registry.^3^ This missingness has been followed by the development of multiple methods of imputation to estimate the missing receptor status before calculating incidence rates and survival estimates.^4,5^

**Figure.**
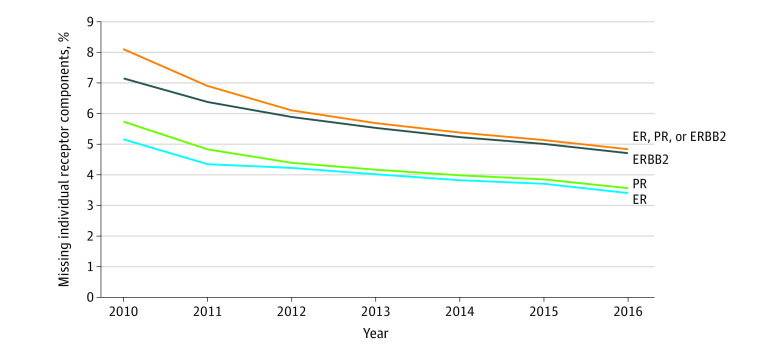
Percentage of Missing Individual Receptor Components in Patients With Invasive Breast Cancer by Year of Diagnosis The percentage of female patients with invasive breast cancer who were missing individual receptor components decreased with year of diagnosis. From 2012 through 2016, the proportion of missing *ERBB2* status was approximately 63% higher than the proportion of missing estrogen receptor (ER) and progesterone receptor (PR).

From a public health perspective, it is essential that all individuals with a diagnosis of invasive breast cancer are tested for ER, PR, and *ERBB2* receptor status because it is critical in making the best decisions regarding the most appropriate systemic and locoregional therapy choices and because it provides prognostic information.^6^ Missing and unknown receptor status have been discussed in several articles.^3,4,5,7,8,9^ However, to our knowledge, this study is the first to describe the population of women with invasive breast cancer reported to SEER with no record of being tested for ER, PR, or *ERBB2* (missing components of receptor status [MCRS]).

## Methods

### Study Population

The SEER Program’s collection of 18 registries (based on the November 2018 submission that was released on April 14, 2019) was used for our analyses. SEER’s population-based cancer registries have a very high estimated completeness of reporting.^10^ Registries used in these analyses cover approximately 27.8% of the US population.^11^ The variables required to identify or distinguish whether hormone receptor tests (ER, PR, or *ERBB2*) were not completed (as opposed to being done but with unknown results) were only available for cases diagnosed through 2017. However, data through 2016 were used so that the type of insurance and urban/rural county of residence could be included in this investigation, as these variables were not available for all data starting in 2017 due to changes in variables included in the SEER data sets. After excluding cases diagnosed through death certificate only and autopsy only, a total of 321 913 women with a diagnosis of invasive breast cancer (*International Classification of Disease for Oncology, Third Edition *[*ICD 0-3*] codes C50.0-C50.9)^12^ from January 1, 2012, to December 31, 2016, were identified. All data used were publicly available, deidentified, and exempted from institutional review board approval and informed consent requirements. The Strengthening the Reporting Observational Studies in Epidemiology (STROBE) reporting guidelines were followed.

A case listing of the study population was generated using SEER*Stat statistical software, version 8.3.5.^13^ The outcome of interest, MCRS, was defined as the breast cancer case missing information about 1 or more of the 3 receptors. This was determined using the following SEER-specific variables: collaborative stage (CS) site-specific factor 1 (ER), CS site-specific factor 2 (PR), and CS site-specific factor 15 (*ERBB2*). Because this study focused on those not being tested, the outcome was defined as an event (MCRS) if any of these 3 site-specific factors was classified as either of the following: 998 (test[s] not done [test(s) not ordered and not performed]) or 999 (unknown or no information; not documented in patient record). Cases in which the tests were ordered but results were not in the medical record or were not interpretable (996: test ordered, not interpretable; 997: test ordered, not in chart) were not considered MCRS.

Common patient and disease characteristics, as well as urban/rural county of residence, were examined for their associations with MCRS. Age at diagnosis was categorized as younger than 50 years, 50 to 64 years, 65 to 79 years, and 80 years or older. Race was identified and reported in the SEER data as the following 5 categories: American Indian or Alaska Native, Asian or Pacific Islander, Black, White, and unknown. Stage at diagnosis was classified as local, regional, distant, and unknown stage/unstaged based on the SEER summary stage. Insurance status at diagnosis categories were insured, insured/no specifics, any Medicaid, uninsured, and insurance status unknown. County-based rural-urban continuum codes were used to classify each case as urban or rural.^14^ These codes broadly described counties as metropolitan or nonmetropolitan. For these analyses, rural-urban continuum codes codes 1 through 3 were classified as urban and 4 through 9 as rural, as has been reported in other studies.^15,16,17,18^ The type of reporting source included 6 categories: hospital inpatient/outpatient or clinic, laboratory only (hospital or private), nursing/convalescent home/hospice, other hospital outpatient unit or surgery center (2006+), physician office/private medical practitioner, and radiation treatment or medical oncology center (2006+).

### Statistical Analyses

Trends in the percentage of missing ER, PR, and *ERBB2* status as well as MCRS were examined by year from 2010 to 2016. All other comparisons were based on the analysis cohort comprising invasive female individuals with breast cancer who received a diagnosis from 2012 to 2016, as these years more accurately depicted the current state of missing ER, PR, and *ERBB2 *status. Descriptive statistics for demographic and clinic characteristics were compared between those with complete receptor designation and those MCRS using χ^2^ tests. All variables of interest were included in a multivariable logistic regression model to identify characteristics and populations with a higher odds of MCRS. The final analyses were completed in February 2022. Analyses were conducted using either SAS, version 9.4 (SAS Institute) or Stata, version 14.2 (StataCorp, College Station, TX). An α of .05 was used to determine statistical significance.

## Results

### Demographic and Clinical Characteristics Overall and by MCRS Status

The analysis cohort included 321 913 female patients with invasive breast cancer who received a diagnosis between 2012 and 2016. Most of the women were 50 years or older (260 077 [81%]), White (252 447 [78%]), with local stage at diagnosis (208 032 [65%]), and insured (228 357 [84%]). More than 90% (302 638) of the cases were reported to SEER from a hospital (inpatient or outpatient) or clinic (Table 1). A significant difference was found between those with MCRS and those with known receptor status for each demographic and clinical characteristic. Notable differences included the following groups with a higher proportion MCRS: 80 years of older, Black or unknown race, distant or unknown stage at diagnosis, reporting sources other than hospital or clinic, and unknown insurance status (Table 1).

**Table 1.** Demographic and Clinical Characteristics by MCRS in 321 913 Female Patients With Invasive Breast Cancer^a^^,^^b^

Characteristic	Total (n = 321 913), No. (%)	Not MCRS (n = 306 663), No. (%)	MCRS (n = 15 250), No. (%)
Age at diagnosis, y			
<49	61 836 (19)	59 313 (19)	2523 (17)
50-64	119 582 (37)	114 562 (37)	4966 (33)
65-79	105 703 (33)	101 031 (33)	4672 (31)
≥80	34 846 (11)	31 757 (10)	3089 (20)
Race			
American Indian or Alaska Native	1928 (1)	1820 (1)	108 (1)
Asian or Pacific Islander	28 173 (9)	26 889 (9)	1284 (8)
Black	36 357 (11)	34 447 (11)	1910 (13)
White	252 447 (78)	240 933 (79)	11 514 (76)
Unknown	3008 (1)	2574 (1)	434 (3)
SEER summary stage			
Local	208 032 (65)	200 195 (65)	7837 (51)
Regional	89 819 (28)	87 671 (29)	2148 (14)
Distant	18 854 (6)	16 538 (5)	2316 (15)
Unknown/unstaged	5208 (2)	2259 (1)	2949 (19)
Type of reporting source			
Hospital inpatient/outpatient or clinic	302 638 (94)	289 783 (94)	12 855 (84)
Laboratory only (hospital or private)	4256 (1)	3388 (1)	868 (6)
Nursing/convalescent home/hospice	223 (0)	41 (<1)	182 (1)
Other hospital outpatient unit or surgery center (2006+)	8283 (3)	7946 (3)	337 (2)
Physicians office/private medical practitioner	3071 (1)	2191 (1)	880 (6)
Radiation treatment or medical oncology center (2006+)	3442 (1)	3314 (1)	128 (1)
Insurance			
Insured	228 357 (71)	220 032 (72)	8325 (55)
Insured/no specifics	41 994 (13)	39 653 (13)	2341 (15)
Any Medicaid	37 486 (12)	35 439 (12)	2047 (13)
Uninsured	5032 (2)	4655 (2)	377 (2)
Insurance status unknown	9044 (3)	6884 (2)	2160 (14)
Urban/rural			
Urban	290 145 (90)	276 600 (90)	13 545 (89)
Rural	31 391 (10)	29 706 (10)	1685 (11)
Unknown/missing/no match	377 (<1)	357 (<1)	20 (<1)

Abbreviations: MCRS, missing components of receptor status; SEER, Surveillance, Epidemiology, and End Results; SSF, site-specific factor.

^a^ Missing is defined as the test not done (SSF code 998) or unknown/no information (SSF code 999).

^b^ A significant difference was found between those missing designation and those not for each demographic and clinical characteristics.

### Percentage of MCRS Within Categories by Demographic and Clinical Characteristics

Overall, 15 250 cases (4.7%) were MCRS. Further examination of MCRS (Table 2) showed higher percentages MCRS in those 80 years or older (3089 [8.9%]), those with unknown race (434[14.4%]), and those with distant stage or unknown/unstaged at diagnosis (2949 [12.3%] and 2949 [56.6%], respectively). Among cases reported by nursing/convalescent homes/hospices, 182 (81.6%) were MCRS, while cases reported by physician offices were 880 (28.7%) MCRS and 868 (20.4%) were MCRS in laboratory only cases. Instances of MCRS were highest for those with unknown insurance status (2160 [23.9%]), followed by those uninsured (377 [7.5%]). Among patients from rural counties, 1685 (5.4%) had MCRS, while 13 545 patients (4.7%) from urban counties had MCRS.

**Table 2.** Percentage of MCRS for Each Category of Demographic and Clinical Characteristics Among Female Patients With Invasive Breast Cancer^a^

Characteristic	MCRS in each category, No (%)
Overall MCRS from 2012-1016 (n = 321 913)	15 250 (4.7)
Year of diagnosis	
2012	3414 (5.5)
2013	3218 (6.1)
2014	3040 (4.7)
2015	2926 (4.4)
2016	2652 (4.0)
Age at diagnosis, y	
<49	2523 (4.1)
50-64	4966 (4.2)
65-79	4672 (4.4)
≥80	3089 (8.9)
Race	
American Indian/Alaska Native	108 (5.6)
Asian or Pacific Islander	1284 (4.6)
Black	1910 (5.3)
White	11 514 (4.6)
Unknown	434 (14.4)
SEER summary stage	
Local	7837 (3.8)
Regional	2148 (2.4)
Distant	2316 (12.3)
Unknown/unstaged	2949 (56.6)
Type of reporting source	
Hospital inpatient/outpatient or clinic	12 855 (4.3)
Laboratory only (hospital or private)	868 (20.4)
Nursing/convalescent home/hospice	182 (81.6)
Other hospital outpatient unit or surgery center (2006+)	337 (4.1)
Physicians office/private medical practitioner)	880 (28.7)
Radiation treatment or medical oncology center (2006+)	128 (3.7)
Insurance	
Insured	8325 (3.7)
Insured/no specifics	2341 (5.6)
Any Medicaid	2047 (5.5)
Uninsured	377 (7.5)
Insurance status unknown	2160 (23.9)
Urban/rural	
Urban	13 545 (4.7)
Rural	1685 (5.4)
Unknown/missing/no match	20 (5.3)

Abbreviations: MCRS, missing components of receptor status; SEER, Surveillance, Epidemiology, and End Results; SSF, site-specific factor.

^a^ Missing is defined as the test not done (SSF code 998) or unknown/no information (SSF code 999).

### Multivariable Logistic Regression

As shown in Table 3, the associations between MCRS and factors of interest were found after adjusting for one another in a multivariable logistic regression model. The adjusted odds of MCRS among those 80 years and older was 1.75 times that of those younger than 49 years. Compared with White women, Black women had higher adjusted odds of MCRS (adjusted odds ratio [aOR], 1.09; 95% CI, 1.04-1.16). Although a high percentage (14.4%) of those with unknown race had MCRS, those with unknown race had lower adjusted odds of MCRS compared with White individuals (aOR, 0.79; 95% CI, 0.68-0.91) which was due to small cell counts when race was combined with other variables in the model. Compared with those with local stage at diagnosis, the adjusted odds of MCRS for those with regional stage was 0.62 (95% CI, 0.60-0.65), while the adjusted odds of those with distant stage and unknown stage or unstaged were more than 3 and 19 times that of local stage at diagnosis, respectively. With hospital inpatient/outpatient or clinic as the reference group, cases reported by laboratory only, nursing/convalescent home/hospice, and a physician’s office were more likely to have MCRS (aOR, 1.42; 95% CI, 1.28-1.60; aOR, 9.37; 95% CI, 6.03-14.53; and aOR, 2.32; 95% CI, 2.06-2.62; respectively). The adjusted odds of MCRS was lower for those with any Medicaid coverage compared with those insured, but was higher for categories of insured/no specifics and insurance status unknown. The adjusted odds of MCRS was higher in rural areas compared with urban areas (aOR, 1.08; 95% CI, 1.03-1.15).

**Table 3.** Adjusted Odds of MCRS Among Female Patients With Invasive Breast Cancer^a^

Level	aOR (95% CI)	*P* value
Age at diagnosis, y		
<49	1 [Reference]	NA
50-64	0.99 (0.94-1.05)	.78
65-79	1.04 (0.99-1.10)	.12
≥80	1.75 (1.65-1.88)	<.001
Race		
American Indian/Alaska Native	1.16 (0.93-1.46)	.18
Asian or Pacific Islander	1.03 (0.97-1.09)	.34
Black	1.09 (1.04-1.16)	.001
White	1 [Reference]	NA
Unknown	0.79 (0.68-0.91)	.001
SEER summary stage		
Local	1 [Reference]	NA
Regional	0.62 (0.60-0.65)	<.001
Distant	3.33 (3.17-3.50)	<.001
Unknown/unstaged	19.39 (18.15-20.72)	<.001
Type of reporting source		
Hospital inpatient/outpatient or clinic	1 [Reference]	NA
Laboratory only (hospital or private)	1.42 (1.28-1.60)	<.001
Nursing/convalescent home/hospice	9.37 (6.03-14.53)	<.001
Other hospital outpatient unit or surgery center (2006+)	0.92 (0.82-1.03)	.16
Physicians office/private medical practitioner	2.32 (2.06-2.62)	<.001
Radiation treatment or medical oncology center (2006+)	0.78 (0.65-0.94)	.008
Insurance		
Insured	1 [Reference]	NA
Insured/no specifics	1.75 (1.60-1.92)	<.001
Any Medicaid	0.71 (0.68-0.76)	<.001
Uninsured	0.97 (0.91-1.04)	.37
Insurance status unknown	1.27 (1.12-1.43)	<.001
Urban/rural		
Urban	1 [Reference]	NA
Rural	1.08 (1.03-1.15)	.004
Unknown/missing/no match	0.99 (0.60-1.65)	.98

Abbreviations: aOR, adjusted odds ratio; MCRS, missing components of receptor status; NA, not applicable; SEER, Surveillance, Epidemiology, and End Results; SSF, site-specific factor.

^a^ Missing is defined as the test not done (SSF code 998) or unknown/no information (SSF code 999).

## Discussion

This cross-sectional study investigated women with invasive breast cancer who were missing components of receptor status (ER, PR, or *ERBB2*) by analyzing information about 321 913 cases from the population-based SEER 18 database. We identified several subgroups of women with higher percentages of MCRS, including older women; Black and American Indian/Alaska Native women and women with unknown race; those with a diagnosis of distant stage or unknown stage/unstaged at diagnosis; those reported from nursing/convalescent homes/hospices, physician offices, or laboratory only; those with unknown insurance status or who were uninsured; and those in rural counties. Differences in MCRS were also found between the categories of subgroups of women in a multivariable analysis. For example, the odds of MCRS increased with age and advancing stage. The odds of MCRS were higher for Black women compared with White women and rural compared with urban residence. These findings were similar to those reported by Howlader et al,^4,19^ although their definition of unknown cases was broader than that used in this study in that they included unknown results and missing data. In addition, the odds of MCRS were typically higher for women who had missing or unknown characteristics. These findings were similar to findings regarding factors associated with unknown stage, unknown grade, and/or incomplete records in central cancer registries, including SEER Program registries.^20^

The importance of establishing the receptor status in patients with breast cancer has been shown in previous studies. Several studies have reported that breast cancer survival varies by molecular subtype.^21,22,23,24^ Hwang et al,^25^ Howlader et al,^19^ and Leone et al^24^ reported that the subtype was a significant factor in breast cancer–specific and overall survival that were also based on SEER data.

Although the importance of testing has been established, some patients with severe comorbidities, deconditioning, and very poor prognosis may not need this testing, as it could be irrelevant for a patient who is only a candidate for best supportive care and hospice. These patients are typically older, with late or unknown stage, and reported by nonhospital facilities. However, for most patients with breast cancer, knowing the receptor status in breast cancer is imperative in determinin the best course of treatment for most other patients. Therapeutic trends have increasingly depended on selecting the most appropriate patients for particular systemic treatments based on a cancer’s molecular makeup. Such treatments include endocrine therapy, small molecule tyrosine kinase inhibitors, chemotherapy, target-specific antibodies, antibody drug conjugates, immunotherapeutic agents, and cellular therapies. More often, such therapies are moving the needle of survival and quality of life. However, patients lacking critical biomarker information cannot benefit from use of these treatments. Conversely, therapeutic approaches that are used inappropriately can be associated with unnecessary harm, low efficacy, and excessive financial burden on patients and the health care system.

### Limitations

Although the results of the present study are potentially informative, some limitations should be considered. Our efforts are limited by the nature of the SEER data, which are deidentified and registry based, and these data do not include information about individual socioeconomic status and comorbidities, both of which may be associated with MCRS. The accuracy of MCRS could not be assessed due to the use of registry data; thus, some misclassification bias likely occurred. In addition, although insurance at the time of diagnosis was examined, there was no available information concerning changes in insurance status during the duration of treatment or details on the cost of testing throughout various areas of the US, and the associated financial burden of testing to individual women could not be analyzed. These factors may be barriers to testing. Lastly, from a public health perspective, we were interested in examining which factors were associated with missing values of receptor status. In this context, they could have been missing either because they was not ordered or for unknown reasons, and combining these 2 may have limited our capacity to make conclusions about whether the test was ordered.

## Conclusions

The results of this cross-sectional study suggest that classifying invasive breast cancer by molecular subtypes based on hormone receptor and *ERBB2* overexpression has been associated with several advancements in diagnosis and effective treatment in women as well as substantial improvements in quality of care and survival outcomes. Despite this testing being recommended by all expert breast cancer guidelines, this study showed that MCRS is still occurring, especially in some socioeconomic populations. The results of this study may help clinicians, public health practitioners, and policymakers target affected populations to minimize or eliminate this critical health disparity and help save more lives.

## Notes

Supplement.
Data Sharing Statement
