# Supplementary material for: Missing Components of Receptor Status Among Women With Invasive Breast Cancer
Source: JAMA Netw Open. 2023 Aug 24;6(8):e2330791. doi: 10.1001/jamanetworkopen.2023.30791 (PMC10450568; doi:10.1001/jamanetworkopen.2023.30791)
Supplement: Supplement. — Data Sharing Statement [file jamanetwopen-e2330791-s001.pdf]

Data Sharing Statement

Stephens. Missing Components of Receptor Status among Women with Invasive Breast Cancer. *JAMA Netw Open*. Published August 24, 2023.
doi:10.1001/jamanetworkopen.2023.30791

Data
Data available: Yes
Data types: Deidentified participant data
How to access data: These authors do not have the permission to share these data. However, they are available upon request from SEER: How to Request Access to SEER Data - SEER Datasets (cancer.gov). (https://seer.cancer.gov/data/access.html)
When available: With publication

Supporting Documents
Document types: None

Additional Information
Who can access the data: Anyone requesting data from SEER, with approval from SEER.
Types of analyses: None
Mechanisms of data availability: With permission from SEER
Any additional restrictions: None
